# Safety evaluation of a recombinant plasmin derivative lacking kringles 2–5 and rt-PA in a rat model of transient ischemic stroke

**DOI:** 10.1186/2040-7378-4-10

**Published:** 2012-05-16

**Authors:** R Christian Crumrine, Victor J Marder, G McLeod Taylor, Joseph C LaManna, Constantinos P Tsipis, Valery Novokhatny, Philip Scuderi, Stephen R Petteway, Vikram Arora

**Affiliations:** 1 Research and Pre-clinical Development, Grifols Therapeutics, Inc., Research Triangle Park, North Carolina, USA; 2 Division of Hematology/Medical Oncology, Department of Medicine, David Geffen School of Medicine at UCLA, Los Angeles, CA, USA; 3 Department of Physiology and Biophysics, Case Western Reserve University, Cleveland, OH, USA

**Keywords:** Ischemic stroke, Δ(K2-K5) plasmin, Intracranial hemorrhage, Spontaneously hypertensive rat model, Recombinant tissue-type plasminogen activator (rt-PA), Middle cerebral artery occlusion (MCAo)

## Abstract

**Background:**

Tissue type plasminogen activator is the only approved thrombolytic agent for the treatment of ischemic stroke. However, it carries the disadvantage of a 10-fold increase in symptomatic and asymptomatic intracranial hemorrhage. A safer thrombolytic agent may improve patient prognosis and increase patient participation in thrombolytic treatment. A novel direct-acting thrombolytic agent, Δ(K2-K5) plasmin, promising an improved safety profile was examined for safety in the snare ligature model of stroke in the rat.

**Methods:**

Male spontaneously hypertensive rats were subjected to 6 hours middle cerebral artery occlusion followed by 18 hours reflow. Beginning 1 minute before reflow, they were dosed with saline, vehicle, Δ(K2-K5) plasmin (0.15, 0.5, 1.5, and 5 mg/kg) or recombinant tissue-type plasminogen activator (10 and 30 mg/kg) by local intra-arterial infusion lasting 10 to 60 minutes. The rats were assessed for bleeding score, infarct volume, modified Bederson score and general behavioral score. In a parallel study, temporal progression of infarct volume was determined. In an in vitro study, whole blood clots from humans, canines and rats were exposed to Δ(K2-K5). Clot lysis was monitored by absorbance at 280 nm.

**Results:**

The main focus of this study was intracranial hemorrhage safety. Δ(K2-K5) plasmin treatment at the highest dose caused no more intracranial hemorrhage than the lowest dose of recombinant tissue type plasminogen activator, but showed at least a 5-fold superior safety margin. Secondary results include: temporal infarct volume progression shows that the greatest expansion of infarct volume occurs within 2–3 hours of middle cerebral artery occlusion in the spontaneously hypertensive rat. A spike in infarct volume was observed at 6 hours ischemia with reflow. Δ(K2-K5) plasmin tended to reduce infarct volume and improve behavior compared to controls. In vitro data suggests that Δ(K2-K5) plasmin is equally effective at lysing clots from humans, canines and rats.

**Conclusions:**

The superior intracranial hemorrhage safety profile of the direct-acting thrombolytic Δ(K2-K5) plasmin compared with recombinant tissue type plasminogen activator makes this agent a good candidate for clinical evaluation in the treatment of acute ischemic stroke.

## Background

Recanalization is an effective treatment for acute ischemic stroke [1]. Currently, recombinant tissue-type plasminogen activator (rt-PA) is the only FDA-approved pharmacological thrombolytic agent for recanalization therapy. Although effective, a major drawback of rt-PA treatment is a 10-fold increase in the risk of symptomatic and asymptomatic intracranial hemorrhage (ICH) [1,2]. A novel thrombolytic agent that effectively lyses clots while significantly reducing the risk of hemorrhage may improve overall prognosis and potentially allow more patients with stroke to be treated.

Recently, a novel recombinant derivative of human plasmin, Δ(K2-K5) plasmin (TAL6003), was produced in an *E. coli* expression system [3]. Δ(K2-K5) plasmin lacks the middle portion (kringles 2–5) of native plasmin and kringle 1 (K1) is spliced directly to the protease domain. As the K1 domain contains the fibrin and the α_2_-antiplasmin (α_2-_AP) binding sites, Δ(K2-K5) plasmin retains all of the fibrinolytic activity as well as the fibrin binding and systemic inhibitory characteristics of native plasmin [3,4]. Δ(K2-K5) plasmin is rapidly neutralized by endogenous inhibitors after IV administration, accounting for its hemorrhagic safety profile. However, this attribute requires Δ(K2-K5) plasmin to be administered by a catheter advanced to the immediate proximity of the thrombus [5-7]. Δ(K2-K5) plasmin shows remarkable hemostatic safety in a rabbit fibrinolytic hemorrhage model [4,6-8], suggesting that, like native plasmin, Δ(K2-K5) plasmin poses significantly less systemic hemorrhagic risk than rt-PA.

In a safety study, a range of test article (compound under study) dosages spanning the presumptive therapeutic dose are selected to determine a No Observable Adverse Effect Level (NOAEL). From these, a margin of safety (NOAEL ÷ therapeutic dose) can be calculated. In this study, a wide dose range of Δ(K2-K5) plasmin (0.15 to 5 mg/kg) spanning the presumptive therapeutic dose of 1 mg/kg, extrapolated from Marder et al. [9] and Crumrine et al. [10], was investigated. The FDA approved thrombolytic agent, rt-PA, was used as an industry standard for comparison as well as a positive control for ICH detection in the model system. Previously, we showed that dosages of rt-PA of less than or equal to 10 mg/kg delivered intra-arterially (IA) caused no more ICH than saline; suggesting a margin of safety for rt-PA of approximately 1 following a 6 hour ischemic insult [11].

Treatment with IV rt-PA is effective when administered within 3 hours of stroke onset [1], but this narrow therapeutic window limits patient eligibility for thrombolytic therapy – estimated at only 1–2% [12]. Recently, the therapeutic window for IV rt-PA treatment was extended to 4.5 hours [2,13] and there is an ongoing clinical study aimed at further extending this window to 6 hours [14]. In light of this, we used a 6 hour ischemic duration in this study to simulate the extended ischemic duration for patient recruitment in future clinical studies – being a safety study, we wanted to mimic the “worst case scenario” for patient studies.

We used the snare ligature model of mechanical middle cerebral artery occlusion (MCAo) in the spontaneously hypertensive rat (SHR) [11] to mimic recanalization therapy for this study based on the remarkable consistency of the SHR in this model system and the necessity to strictly control the ischemic duration. The latter is not possible with a thromboembolic model where the onset of rt-PA treatment can be controlled but the onset of recanalization cannot. For example, treatment with rt-PA 1 hour after cerebral thrombosis in the rat results in recanalization 15 to 60 minutes later, making the effective ischemic duration between 75 and 120 minutes. In our previous study, we showed that ischemic duration profoundly affects infarct volume. In a safety study such as this, uncontrolled recanalization would make the infarct volume data uninterpretable. This would also be true for the hemorrhage data as ICH liability increases with increased ischemic duration [11]. Furthermore, control groups such as saline and vehicle treatment in a thromboemoblic model would necessarily reflect permanent occlusion as neither can lyse a clot. These complications of a thromboembolic model would prevent the establishment of a true baseline, leading to distortion and misinterpretation of the data. In addition, the snare ligature model of MCAo allowed for the initiation of IA treatment to immediately precede reflow, better reflecting thrombolytic treatment in humans. This is not possible in an intralumenal model as there would be a significant time delay to replace the occluder with a dosing catheter; this is in addition to other complications of this model [15,16]. Although the snare ligature model is somewhat obscure, it is well characterized; having been used to study high energy metabolites [17-19], temporal infarct volume progression [20], protein kinase C activity [21] and in MRI studies [22].

We used the SHR in our study because of the remarkable consistency of this strain in stroke studies [11,23]. Furthermore, the strain has a relevant comorbidity for stroke, hypertension, fulfilling a Stroke Therapy Academic Industry Roundtable (STAIR) recommendation for drug development for a stroke indication [24] and thus may be more relevant for human translational studies [23]. Additionally, the SHR is resistant to experimental therapeutic stroke paradigms [25] (personal experience) with the exception of reflow, very similar to humans. Finally, an ischemic duration of 6 hours was selected to simulate an extended interval between stroke symptom onset to treatment in the clinic.

In summary, as the in vivo thrombolytic efficacy of Δ(K2-K5) plasmin has been established in large animal models of arterial thrombosis, the primary focus of this study was to assess the ICH safety of Δ(K2-K5) plasmin administered after an extensive cerebral ischemic insult with recanalization. rt-PA was used as an industry standard and to validate the ability of the model to detect ICH. In this report, we established a margin of safety for rt-PA after 6 hours ischemia with reflow and compared it to the margin of safety of Δ(K2-K5) plasmin.

## Methods

### In vitro clot lysis assay

Approximately 87 mg retracted whole venous blood clots from humans, canines and rats were exposed to 1.14 mg/mL Δ(K2-K5) plasmin at a 2:1 volume to weight ratio at room temperature. At designated time points, 5 μL were transferred into 3 ml of 0.9% NaCl and the degree of clot lysis was evaluated by increased absorbance at 280 nm over time. For controls, clots were exposed to equal volumes of saline.

### Animals

Adult male SHRs weighing 330–380 g were obtained from Charles River Laboratories (Raleigh, NC). Animal experiments were conducted at North Carolina State University (NCSU) College of Agriculture and Life Sciences (CALS). The animal use protocol was reviewed and approved by the NCSU Institutional Animal Care and Use Committee prior to the initiation of the study and was performed in compliance with standards set forth by the CALS animal facilities.

Upon arrival, the rats were assigned a number by the animal facility staff and housed individually. They were allowed at least one week to acclimate. The rats were on a 12 hour light/dark diurnal cycle with food and water provided ad libitum. To conserve test article, rats were assigned to experimental groups in tandem (2 rats to the same group/experimental day) on a rotating basis as outlined in Table 1.

**Table 1:** Experimental groups and dosing characteristics

**Group**	**n**	**Dose (mg/kg)**	**Test Article Concentration (mg/mL)**	**Injection Volume**	**Infusion Duration**
**(Test Article)**				(μL/g BW*)	
**Saline**	6	Dose Vol	---	1	10 min
**Vehicle**	6	Dose Vol	---	1	
**rt-PA**	5	10	5^†^	2	20 min
	6	30	5^†^	6	60 min
**Δ(K2-K5) Plasmin**	6	0.15	0.3^‡^	0.5	10 min
	6	0.5	0.5	1	
	6	1.5	1.5	1	
	5	5	5	1	

* BW is the body weight. ^†^ this solution was hyperosmotic (792 mOsmol/L). Solutions of higher concentrations and thus higher osmolality may have significant physiological effects in ischemic brain tissue. ^‡^ lower concentrations of Δ(K2-K5) plasmin may have compromised the integrity of the molecule. The total volumes infused averaged 0.35 mL, 0.7 mL and 1.95 mL for infusion durations of 10, 20 and 60 minutes, respectively.

### Transient middle cerebral artery occlusion model

**Figure 1:**
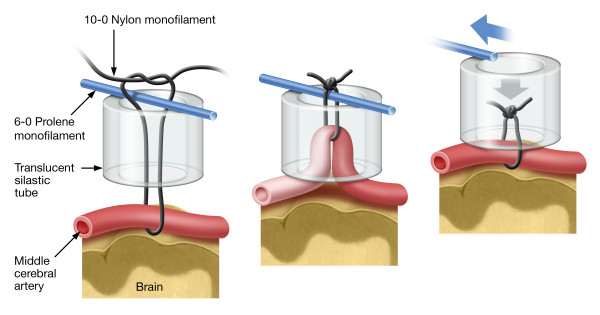
**Rat snare ligature model.** The head of the rat was immobilized in a specially designed head holder. A skin incision was made between the eye and external auditory canal. The MCA was exposed by bisecting the temporalis muscle and performing a craniotomy anteriomedial to the juncture of the zygomatic arch with the squamosal bone. The dura was opened using a tuberculin syringe and the MCA was isolated from the arachnoid and pia by blunt dissection. Left Panel: construction of the snare ligature. Middle Panel: occlusion of the middle cerebral artery. Right Panel: removal of the snare ligature resulting in recanalization.

Six hours of MCAo was produced in the SHR using the snare ligature model first described in the mouse [26], adapted to the rat [11,27] and shown schematically in Figure 1. Briefly, rats were fasted overnight to obtain low, stable plasma glucose concentrations. They were anesthetized with isoflurane (5% in 100% oxygen), orotracheally intubated and administered buprenorphine (0.03 mg/kg, subcutaneously) for pain relief. A tail arterial catheter was placed to monitor blood pressure and to obtain arterial blood samples for blood gas analysis. Following arterial catheterization, artificial ventilation (Model 683 small animal ventilator, Harvard Apparatus, Natick, MA) was initiated. The isoflurane anesthetic gas (1.0–1.5 %) was driven by compressed air supplemented with oxygen to prevent blood hyperoxia. The head of the rat was immobilized in a specially designed head holder. A skin incision was made between the external auditory canal and the eye. The lateral aspect of the skull was exposed by cutdown and a small (~2 mm) craniotomy was performed anteriomedial to the zygomatic arch. The dura was incised and the middle cerebral artery was gently dissected from the arachnoid and pia. A snare ligature device constructed from 10–0 nylon suture, 6–0 prolene suture and a piece of silastic tubing (OD/ID 1.65 mm/0.76 mm; Dow Corning Corporation) (Figure 1, left panel) was used to occlude the left MCA (Figure 1, middle panel). Following MCAo, the rats were recovered from anesthesia, extubated and returned to a clean cage. The rats had free access to food (Hydro Gel and Diet Gel-Recovery; ClearH_2_O, Portland, ME) during the time interval between placement and removal of the snare ligature.

**Figure 2:**
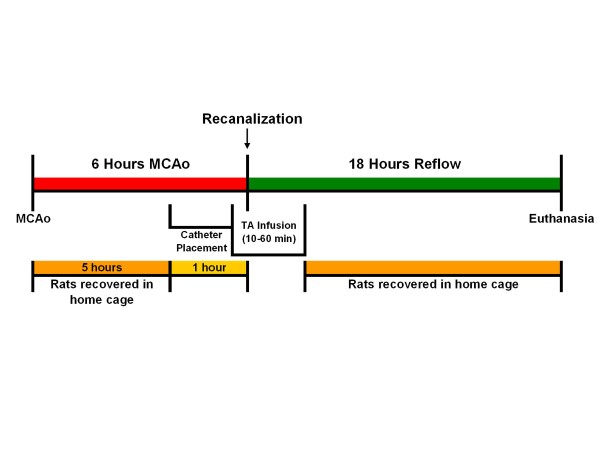
Timeline of the experiment.

During surgery, the blood pressure was continuously recorded electronically using the Ponemah Physiological Monitoring Platform (DSI, Cleveland, OH). Body temperature was maintained at or slightly above 37°C using a heat lamp in a feedback circuit with a rectal temperature probe (TCAT-2 Temperature Controller, Physitemp Instruments, Inc., Clifton, NJ). Blood gas measurements (i-Stat hand-held physiological monitor, Heska AG, Switzerland) were obtained before MCAo and adjustments were made if necessary. Surgical sham animals showed no damage to the brain [11]. For clarity, a timeline of the study is presented in Figure 2.

### Experimental groups and dose administration

**Figure 3:**
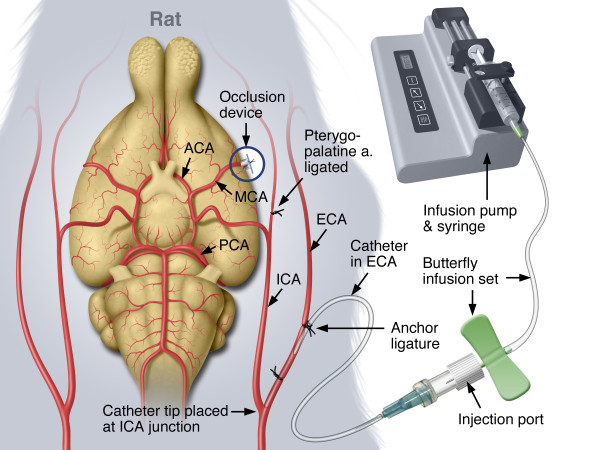
**Dosing of thrombolytic agents.** For clarity, the illustration shows a ventral analogy. In practice, once the EC-ICA was cannulated, the rat was gently rolled onto his right flank and the injection port was secured to the left flank. The head was, again, immobilized in the head holder. During this process, patency of the catheter was maintained by saline infusion (0.75 mL/hr). Infusion of test article was accomplished by replacing the saline flush syringe butterfly assembly with an assembly containing the test article. The infusion of the test article was initiated approximately 1 minute before dismantling the snare ligature while the head was secured in the head holder.

Table 1 shows the experimental groups and the dosing characteristics for each group. The IA dosing technique has been described in detail [11] and is shown in Figure 3. Briefly, approximately 5 hours after MCAo, the rats were assessed for neurological function [11,28], re-anesthetized, intubated and artificially ventilated. The extra-cranial internal carotid artery (EC-ICA) was catheterized via the external carotid artery (ECA) by cutdown (Figure 3). The snare ligature was exposed and test article administration was initiated. Approximately 1 minute later, the snare ligature was dismantled (Figure 1, right panel). The surgical site was flooded with papaverine to dilate the MCA. Recanalization was confirmed by visual inspection of the artery through the surgical microscope (Zeiss OPMI-6 C, Prescott’s Inc., Monument, CO). Parallel control experiments using latex injected into the EC-ICA immediately following reflow showed latex filling of the MCA vascular tree [11] confirming recanalization. Following dosing, the rats were recovered from anesthesia, administered a second dose of buprenorphine (0.03 mg/kg, subcutaneously) and returned to their home cage. EC-ICA dosing shams (dose volume of saline 6 hours after sham MCAo surgery) showed no damage to the brain [11].

All 7 dosing solutions (see Table 1) were prepared in total before beginning the experiment. They were filtered (0.22 μm filter), dispensed into 1 mL aliquots, snap-frozen and stored at −80°C. Saline for injection (filtered and frozen) was used as a control for the vehicle (saline with 2% trehalose, acidified to pH 3.5 with citric acid).

Five vials of lyophilized rt-PA (Alteplase, Genentech, Inc., South San Francisco, CA) were solubilized at 5 mg/mL and pooled before filtering and freezing. rt-PA solution, stored frozen at −80°C, is stable for at least 7 years [29]. Due to solubility and osmolality limitations, dosing concentrations of rt-PA >5 mg/mL were not practical [11]. The 10 mg/kg dose of rt-PA corresponds to the efficacious IV dose in rats which is 10-fold higher than in humans. The discrepancy derives from a 10-fold lower sensitivity of rat clots to lysis by human rt-PA [30].

Test articles were administered by IA infusion at a rate of 0.033–0.040 mL/min (except for the 0.15 mg/kg Δ(K2-K5 plasmin group; see Table 1).

### Bleeding score

Twenty four hours after MCAo onset, the rats were deeply anesthetized with isoflurane (5% in O_2_). The brains were perfused transcardially with heparinzed saline (10 U/mL), removed from the calvarium and immersed in ice cold saline. Digital photographs of the gross brain were obtained.

Sequential 2 mm coronal sections were obtained throughout the neocortex (8–9 sections/brain). The sections were stained with 1–2% 2, 3, 5 triphenyl tetrazolium chloride (TTC) [11,31], digitally photographed with a ruled standard and fixed in 10 % buffered formalin.

Two 10 μm hematoxylin and eosin (H&E) stained sections, spaced 250 μm apart, were obtained from each original 2 mm TTC stained section (16–18; 10 μm sections/rat brain) and evaluated for bleeding (Bleeding Score) using a 7 category scoring system: 0 = Non hemorrhagic ischemic infarction, 1 = Dispersed individual petechiae, 2 = Confluent petechiae, 3 = Hemorrhagic infarction, 4 = Parenchymal hemorrhage extending beyond the infarction, 5 = Death before planned termination due to parenchymal hemorrhage, 5.5 = Hemorrhage in non-ischemic brain tissue (for examples of the Bleeding Score, see Crumrine et al. [11]). The brains were evaluated in half score increments. The overall score for the rat brain was the highest score of the evaluated sections.

Bleeding Score was determined in a blinded fashion by CPT in the laboratory of JCL, Department of Physiology and Biophysics, Case Western Reserve University, Cleveland, Ohio.

### Infarct volume analysis

The digital photographs of the fresh TTC stained brain sections were sent to JCL’s laboratory at Case Western Reserve University in Cleveland, OH. CPT imported each photograph into an image analysis program (Image-Pro Plus v4.5, Media Cybernetics, Inc., Bethesda, MD). The infarct volume, reported in mm^3^, was determined by the indirect method in a blinded manner by CPT.

### Neurological function analysis (modified Bederson score)

The rats were assessed for neurological function twice during the study – once prior to the induction of anesthesia to place the EC-ICA catheter for test article administration and again just before euthanasia. The first assessment was used to screen for stroke presence. Any rat not displaying a score of at least 2 was eliminated from the study. The second score was analyzed as an experimental variable.

A modified Bederson score [11,28] was used with the following definitions: Score 0: No apparent neurological deficits; Score 1: Body torsion present; Score 2: Body torsion with right side weakness; Score 3: Body torsion, right side weakness with circling behavior; Score 4: Seizure Activity.

The score was assessed in a non-blinded manner by Grifols Therapeutics, Inc. personnel (RCC or GMT).

### General behavioral score

In addition to the neurological score, a general behavioral score was developed. The score was used to assess the general alertness and responsiveness of the rats 24 hours after MCAo; behaviors not captured in the modified Bederson score. The score had the following definitions: Score 0: Behavior consistent with a normal naïve rat (i.e. no ipsilateral deficit); Score 1: Bright/active/responsive; the rat spontaneously moves and explores his cage, responds to external stimuli, explores the top of the cage; Score 2: Quiet/alert/responsive; reserved behavior but will respond to external stimulus, tends not to rear or explore the top of the cage; Score 3: Depressed behavior: tends not to move unless prodded, quickly returns to a somnolent state, little to no interest in external stimuli; Score 4: Unresponsive: remains in a prostrate position even when prodded; Score 5: Seizure activity requiring euthanasia.

The behavioral categories were developed by the CALS personal for the purpose of monitoring recovery of animals following surgical procedures (standard CALS post-operative care). A numerical value was assigned to the predetermined behavioral observations. The rats were scored by the same person (BJW) from CALS in a blinded manner.

### Exclusion criteria

Animals were excluded from the study for the following reasons: breakage of the MCA during occlusion or recanalization; breakage of a side branch of the MCA causing bleeding at the surgical site; lack of right side weakness; occlusion device failure; reflow could not be visually confirmed; bruising of the brain during the occlusion surgery; air bubbles or particles in the ICA catheter during infusions; surgical complications requiring euthanasia, evidence of bleeding upon reopening of the surgical site, plasma glucose concentration >11.5 mmol/L prior to MCAo.

### Statistics

Analysis of the infarct volume and physiological variables between the experimental groups was accomplished by ANOVA followed by Tukey-Kramer HSD multiple comparison test (JMP statistical software, SAS Institute, Inc., Cary, NC).

Statistical analysis of the modified Bederson score and the general behavioral score was accomplished using the Kruskal-Wallis one way ANOVA followed by Newman-Keuls multiple comparisons test for non-parametric data using the GBstat statistical package (Dynamic Microsystems, Inc., Silver Spring, MD).

To estimate the number of animals per group, a power analysis was performed on the vehicle group from a contemporary pilot study using infarct volume as the test variable. To observe a statistically significant difference of 30% between means of the experimental groups at an α level of 0.05 and a β level of 0.8, a minimum of 5 animals would be required (personal observation). This is consistent with Brint et al. [23] and with our past experience using the SHR in this model system.

## Results

### In vitro thrombolysis

**Figure 4:**
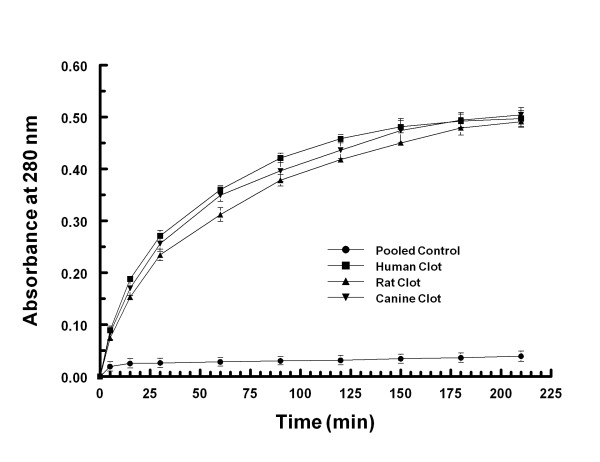
**Clot lysis kinetics of Δ(K2-K5) plasmin.** Retracted whole blood clots (87 mg) from humans, canines and rats were exposed to 1.14 mg/mL Δ(K2-K5) plasmin in vitro. At the indicated time points, samples were assayed for optical density at 280 nm.

No change in absorbance at 280 nm was observed when retracted whole blood clots were exposed to vehicle (Figure 4). Clots from humans, canines and rats showed nearly identical lysis kinetics when exposed to Δ(K2-K5) plasmin (Figure 4).

### Exclusion of animals and physiological variables

Forty eight rats were assigned to the experimental groups. Ten were excluded from the study based on the a priori exclusion criteria including: breakage of the MCA (2), excessive surgical bleeding (3), lack of right side weakness (1), missing brain sections (2), high pre-ischemia plasma glucose concentration (1), and non-recovery from anesthesia (1).

The physiological variables prior to MCAo, prior to reflow and just after completion of test article infusion are presented in Additional file 1 (Tables S1, Additional file 1: S2, and Additional file 1: S3). All of the variables were within physiological range. There was a marked increase in the plasma glucose concentrations prior to reflow which reflect the rats having free access to food between the two procedures (comparison of glucose values in Table S1 and S2). There was a significant increase in the pulse pressure following infusion of 10 and 30 mg/kg of rt-PA as compared to the pre-reflow values (TAL6003-Suppl-RCC; comparison of values in Table S2 and S3).

### Intracranial hemorrhage

**Figure 5:**
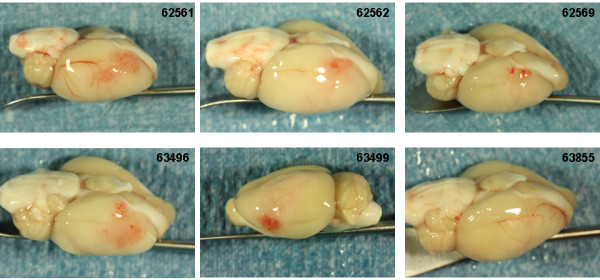
**Photographs of the brains from the vehicle group. A**) Photographs of the gross brain of each rat. **B**) Representative TTC Stained Sections from each rat. Small blue arrows indicate areas of petechial hemorrhage.

**Figure 6:**
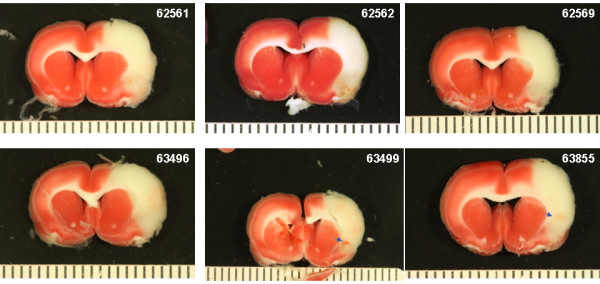
**Brain photographs from the 10 mg/kg rt-PA group. A**) Photographs of the gross brain of each rat. **B**) Representative TTC Stained Sections from each rat. Small blue arrows indicate areas of petechial hemorrhage. Large blue arrows indicate hemorrhagic infarction.

**Figure 7:**
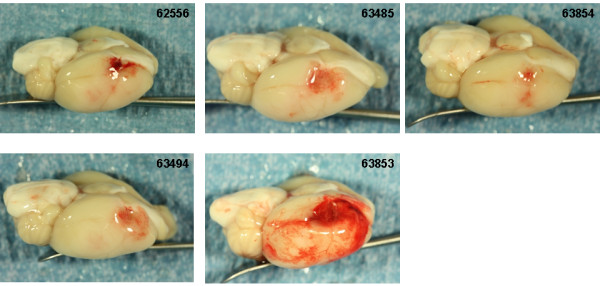
**Brain photographs from the 30 mg/kg rt-PA group. A**) Photographs of the gross brain of each rat. **B**) Representative TTC Stained Sections from each rat. Small blue arrows indicate areas of petechial hemorrhage. Large blue arrows indicate hemorrhagic infarction. Large black arrows indicate hemorrhage to the brain stem (**A**) or damage to the hypothalamus or pons (**B**).

**Figure 8:**
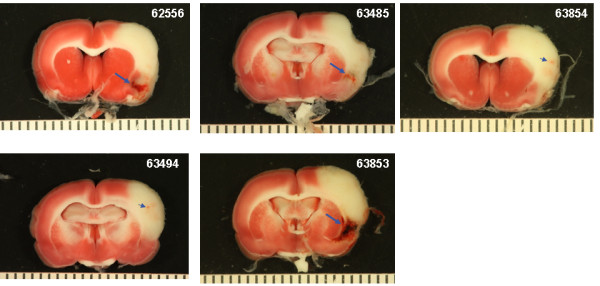
**Brain photographs from the 5 mg/kg Δ(K2-K5) plasmin group. A**) Photographs of the gross brain of each rat. **B**) Representative TTC Stained Sections from each rat. Small blue arrows indicate areas of petechial hemorrhage.

Photographs of the gross brain and representative TTC stained 2 mm sections from rats treated with vehicle, 10 and 30 mg/kg rt-PA and 5 mg/kg Δ(K2-K5) plasmin are shown in Figures 5, 6, 7, and 8. Vehicle treated rats had some superficial bleeding in the gross brain (Figure 5A) but the TTC stained sections are mostly hemorrhage free. However, there was evidence of small areas of petechial hemorrhages in rats 63499 and 63855 (Figure 5B, small blue arrows) which was confirmed by histological evaluation. Rats treated with 10 mg/kg rt-PA showed more bleeding on the brain surface compared to vehicle especially for rat 63853 where blood covered a large portion of the ipsilateral hemisphere (Figure 6A) portending a large parenchymal hemorrhage in the TTC stained section (Figure 6B, large blue arrow). Two animals showed smaller hemorrhagic infarcts (Figure 6B; rats 62556, 63485, large blue arrows) and two others showed evidence of petechial hemorrhage (Figure 6B, small arrows). There were no deaths or premature euthanasia.

Increasing the dose of rt-PA to 30 mg/kg resulted in more severe bleeding (Figure 7). Exclusive to this group was hemorrhage in the brain stem in 3 of 6 rats (Figure 7A and B; rats 62565, 64575, 63856, large black arrows). In two of these (rats 63856, 64575), bleeding in the brain stem was associated with hypothalamic damage and seizure activity, requiring premature euthanasia while the third (62565) had bleeding in the pons area (Figure 7B; large black arrows). Three rats had evidence of hemorrhagic infarction (62559, 62565, 63856; Figure 7B, large blue arrows), one rat showed extensive petechial hemorrhage (62560) and one rat had a seemingly clean infarct (63482). These observations were similar to those noted in a previous study using rt-PA in this model system [11].

Treatment with the highest dose of Δ(K2-K5) plasmin (5 mg/kg) resulted in little to moderate superficial bleeding (Figure 8A). There was evidence of petechial bleeding in three rats (Figure 8B, small blue arrows) and 2 rats appeared to have clean infarcts.

**Figure 9:**
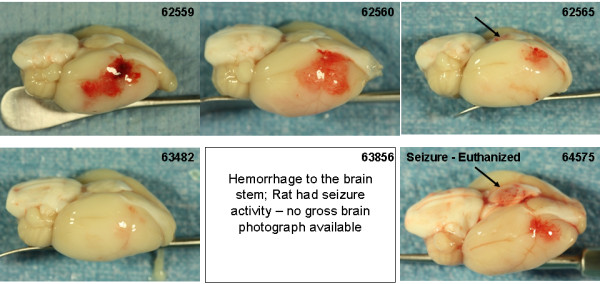
**Box-Whisker Plot of the Bleeding Score.** * p < 0.05 for the indicated groups (Kruskal-Wallis ANOVA, Newman-Keuls multiple comparison test for non-parametric data; n = 6, 6, 5, 6, 6, 6, 6, 5, respectively). Values are the median ± the 5 and 95 percentile.

The Bleeding Score based on histological evaluation of H&E sections as well as gross observations for the groups are presented in Figure 9 (graphed data are presented in Additional file 1, Table S4). Rats treated with saline or vehicle had minimal bleeding. Rats treated with 10 mg/kg rt-PA showed significantly more bleeding than both the saline and vehicle groups. Increasing the dose of rt-PA to 30 mg/kg increased bleeding severity although not to the level of statistical significance compared to the 10 mg/kg rt-PA group (Figure 9).

The Bleeding Scores for all 4 Δ(K2-K5) plasmin treatment groups (Figure 9) were low and similar to those animals treated with saline or vehicle (compare Figure 5 with Figure. 8). On the contrary, the scores for both rt-PA groups were higher than in animals receiving control infusions.

Although Δ(K2-K5) plasmin-treated animals tended to have lower Bleeding Scores than the 10 mg/kg rt-PA group, these differences failed to reach the level of statistical significance. All four Δ(K2-K5) plasmin treated groups had significantly less bleeding compared to the 30 mg/kg rt-PA treated group. There was no evidence of increased bleeding severity with increasing dose of Δ(K2-K5) plasmin (Figure 9).

### Infarct volume

**Figure 10:**
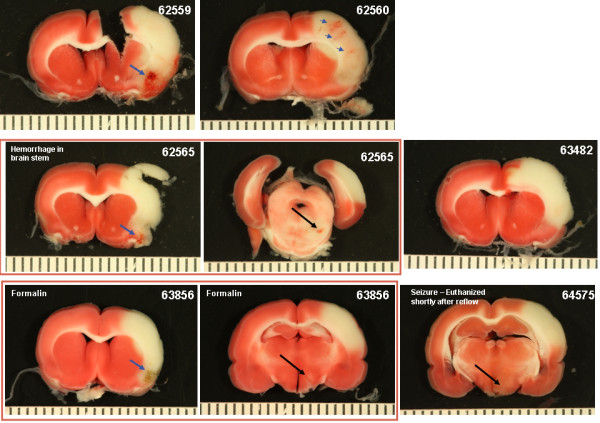
**Temporal progression of the infarct volume.** Rats were exposed to the designated duration of MCAo. All rats were euthanized 24 hours after MCAo onset to allow the infarct to mature. Statistics: Groups with like letters are not significantly different (p < 0.05; ANOVA, Tukey-Kramer HSD). Group values are the mean ± SEM.

The temporal progression of infarct volume development for the SHR in our model is presented in Figure 10. Rats were subjected to 1, 1.5, 2, 4, 6 and 24 hours (permanent) MCAo followed by reflow. All rats were euthanized 24 hours after onset of MCAo. As the ischemic duration was increased from 1 (infarct volume 12 ± 7 mm^3^) to 4 (infarct volume 171 ± 17 mm^3^) hours, there was a progressive increase in infarct volume which was particularly noticeable between 1.5 (55 ± 6 mm^3^) and 2 hours MCAo (158 ± 13 mm^3^); a Δ change of 103 mm^3^. The infarct volume after 2 and 4 hours MCAo was similar to permanent occlusion (166 ± 10 mm^3^) of the MCA. Increasing the ischemic duration to 6 hours (261 ± 20 mm^3^) resulted in significantly larger infarcts than all other groups. Sham animals did not have any observable damage to the brain [11].

The contralateral/ipsilateral hemisphere ratio (indicative of brain swelling) for the 1 hour group was nearly identical to that of the sham animals (0.98 ± 0.02). Animals subjected to 6 hours of MCAo with reflow had greater cerebral swelling than did all other ischemic durations. Animals subjected to 1 hour ischemia had less edema than rats with permanent MCAo (Figure 10).

**Figure 11:**
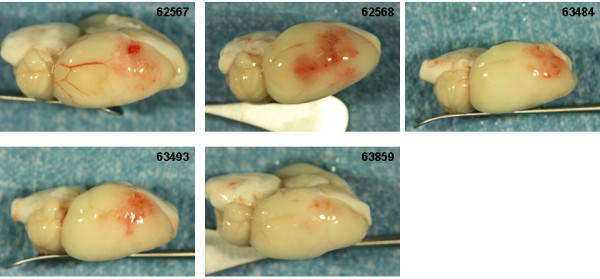
**Scatter plot of the infarct volume of the experimental groups.** Rats were subjected to 6 hours middle cerebral artery occlusion followed by 18 hours reflow. Test articles were infused IA over 10 to 60 minutes beginning 1 minute before reflow. * p < 0.05 for the indicated groups (ANOVA, Tukey Kramer HSD; n = 6, 6, 5, 6, 6, 6, 6, 5, respectively). Group values are the mean ± SEM.

The infarct volumes for the experimental groups in the MCAo safety study are presented in Figure 11. Due to the ischemic duration and the use of the SHR, the scatter plot of the individual animals reveals the remarkable consistency of the infarct data.

The study consisted of 4 control groups. The saline group was a control for the vehicle group. The vehicle group was a control for the Δ(K2-K5) plasmin groups. Both of the rt-PA groups acted as positive controls. The infarct volumes were consistent over the 4 control groups (269 ± 18 mm^3^, 240 ± 17 mm^3^, 255 ± 13 mm^3^, and 258 ± 11 mm^3^, for saline, vehicle and 10 and 30 mg/kg rt-PA groups, respectively). Statistically they were the same (p > 0.95; saline vs. vehicle, p = 0.9043). Rats treated with Δ(K2-K5) plasmin tended to have smaller infarct volumes compared to controls. Rats treated with 0.15 mg/kg Δ(K2-K5) plasmin had a significantly smaller infarct volume (194 ± 18 mm^3^) compared to saline (p = 0.0449). There was a trend (not statistically significant) for a progressive reduction in infarct volume with Δ(K2-K5) plasmin dosages of 0.5 mg/kg (237 ± 15 mm^3^) to 1.5 mg/kg (221 ± 22 mm^3^) to 5 mg/kg (197 ± 12 mm^3^) (Figure 11).

### Modified Bederson score

**Figure 12:**
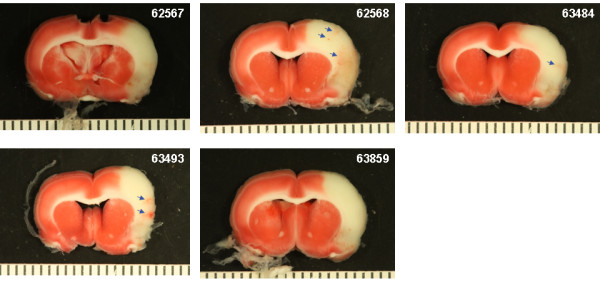
**Modified Bederson score.** Rats were exposed to 6 hours middle cerebral artery occlusion followed by 18 hours reflow. Test articles were infused IA over 10 to 60 minutes beginning 1 minute before reflow . * p < 0.05 compared to all other groups (Kruskal-Wallis, Newman-Keuls; n = 6, 6, 5, 6, 6, 6, 6, 5, respectively). Values are the median ± the 5 and 95 percentile.

The modified Bederson score data are presented in Figure 12 (graphed data are presented in Additional file 1, Table S5). The 30 mg/kg rt-PA group had a significantly worse functional outcome compared to all of the other groups in the study. Rats treated with Δ(K2-K5) plasmin were not significantly different from those receiving saline or vehicle groups.

### Behavioral score

**Figure 13:**
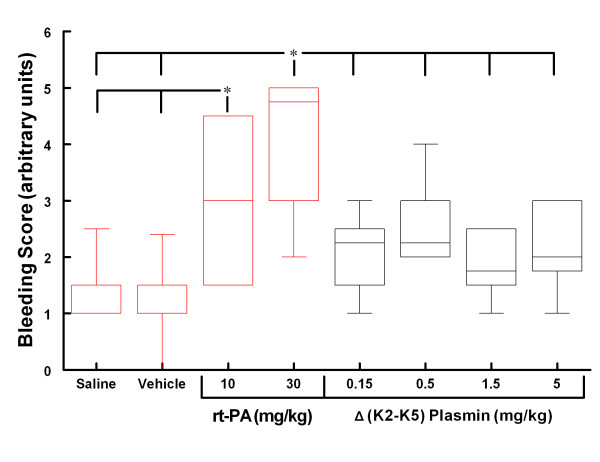
**General behavioral score.** * p < 0.05 compared to all other groups; ^†^ p < 0.05 compared to vehicle; ^‡^p < 0.05 compared to saline (Kruskal-Wallis, Newman-Keuls; n = 6, 6, 5, 6, 6, 6, 6, 5, respectively). Values are the median ± the 5 and 95 percentile.

The general behavioral score data are presented in Figure 13 (graphed data presented in Additional file 1, Table S6). Rats treated with saline, vehicle or 10 mg/kg rt-PA were active and alert after 18 hours of recovery. Rats treated with 30 mg/kg rt-PA were very lethargic characterized by lack of spontaneous movement. In general, rats receiving Δ(K2-K5) plasmin were more alert and active than rats treated with saline, vehicle or rt-PA. Rats dosed with 5 mg/kg Δ(K2-K5) plasmin were significantly more alert and active than saline controls.

## Discussion

As in vivo thrombolytic efficacy has been previously established, the primary focus of this study was to determine the ICH safety liability of the novel direct acting thrombolytic agent Δ(K2-K5) plasmin following an extended ischemic insult in a model of focal cerebral ischemia in the rat. An ischemic duration of 6 hours was selected to better mimic extended symptom onset to treatment in the clinic and to simulate a “worst case scenario” common in safety studies. The main finding of the study was that high dose Δ(K2-K5) plasmin treatment was statistically no worse than low dose rt-PA at causing ICH but had a greater margin of safety. Surprisingly, secondary observations suggested that Δ(K2-K5) plasmin administration tended to reduce infarct volume and significantly improve behavior.

### In vitro efficacy and dose considerations

Our in vitro data (Figure 4) suggests that Δ(K2-K5) plasmin would have equal thrombolytic efficacy in rats, canines, and humans. Indeed, on an equal molar basis, native plasmin/Δ(K2-K5) plasmin demonstrates similar lysis kinetics for clots derived from a host of species including humans, canines, rabbits, rats, bovine, and ovine [3,4,32]. This is in contrast to rt-PA where a 10-fold greater dosage is required for rat vs. human thrombolysis. This likely reflects the difference in the mechanism of action between the two molecules. Δ(K2-K5) plasmin acts directly on clot fibrin whereas rt-PA requires the intermediate step of conversion of plasminogen to plasmin. Rat plasminogen appears to be a 10-fold less sensitive substrate for human rt-PA. Recently, Haeloewyn et al., [33] challenged this conclusion, suggesting that 0.9 mg/kg rt-PA is equally as effective as 10 mg/kg in the rat when treatment was started 45 minutes post MCA thrombosis. Inspection of the data shows that 0.9 mg/kg rt-PA resulted in a significant delay to reflow, significantly reduced cumulative reperfusion, a near doubling in the infarct volume and a near tripling in edema volume compared to 10 mg/kg rt-PA treated animals. The reason the latter two variables were not statistically significantly different between the two groups was due to the large variability in the 10 mg/kg rt-PA group. Wang et al., [34] showed that treatment with 5 mg/kg rt-PA beginning 1 hour following MCA thrombosis in the rat did not reduce infarct volume compared to control whereas 10 mg/kg rt-PA did. Taken together, 10 mg/kg rt-PA is clearly more therapeutic in the rat than 0.9 mg/kg. Our temporal data suggests that the rate of clot lysis following thrombolytic treatment is crucial for improved outcome. This is because the rate of thrombolysis determines the overall ischemic insult and is related to the local concentration of the thrombolytic agent which in turn is related to the dose. This reflects clinical experience where early recanalization is correlated with improved outcome [35,36].

The in vivo thrombolytic efficacy of Δ(K2-K5) plasmin was established in rabbit and canine thrombosis models because larger animals better accommodated the dosing requirements of direct acting thrombolytic agents. In a rabbit model of MCA thrombosis, 0.25 to 1 mg/kg native plasmin lysed the thrombus and restored blood flow to the brain [9]. The molecular weight of Δ(K2-K5) plasmin is approximately half that of native plasmin (37000 vs. 81000, respectively) and because the catalytic properties of native and Δ(K2-K5) plasmin are identical [3,4], on a molar to molar basis, the analogous dose for Δ(K2-K5) plasmin would be 0.114 to 0.5 mg/kg. This efficacious dosage estimate was recently confirmed using this same model system (Marder, personal communication). In a Beagle dog model of femoral artery thrombosis (artery diameter similar to human MCA), the thrombolytic efficacious dose for Δ(K2-K5) plasmin was approximately 1 mg/kg [10]. These data, coupled conservatively with our in vitro data showing equal lytic activity on clots derived from rats, canines and humans (Figure 4), support a thrombolytic efficacious dose of 1 mg/kg Δ(K2-K5) plasmin in the rat.

### Intracerebral hemorrhage – safety

In a safety study, escalating doses of test article are used to establish a margin of safety by relating the efficacious dose to the NOAEL dose (margin of safety = NOAEL ÷ efficacious dose). In this study, the NOAEL dose was defined as the dose at which ICH was no greater than saline and vehicle. We used rt-PA as an ICH positive control. The 30 mg/kg rt-PA dose was clearly toxic and so confirmed that the 10 mg/kg dose, the therapeutic dose in rats, was near the NOAEL dose. Thus, a conservative estimate of the margin of safety for rt-PA at 6 hours cerebral ischemia in the rat is equal to 10 mg/kg (NOAEL dose) ÷ 10 mg/kg (Efficacious dose), or 1. However, because 10 mg/kg rt-PA caused significantly more bleeding than saline and vehicle, the margin of safety, in reality, is less than 1. This result is similar to that observed in an IA dose escalation study using rt-PA (1, 5, 10 and 30 mg/kg) under the same conditions described here [11]. In that study, IA doses of rt-PA less than 10 mg/kg did not result in greater ICH compared to saline; indicating that 10 mg/kg is the highest no ICH effect dose [11]. In contrast, the presumptive thrombolytic efficacious dose for Δ(K2-K5) plasmin is 1 mg/kg based on cerebral thrombolysis studies in the rabbit [9] (Marder, personal communication) and a femoral artery thrombolysis study in the dog [10]. In this investigation, no increase in ICH over control was observed even at doses 5 times greater than the presumptive efficacious dose (5 mg/kg; highest dose tested); indicating a margin of safety of greater than 5. Therefore, Δ(K2-K5) plasmin demonstrates at least a 5-fold greater safety margin than rt-PA following an extended ischemic insult in the rat.

Ischemia predisposes the cerebral vasculature and parenchyma to bleeding complications following reflow, especially after an extended ischemic event [37]. High concentrations of thrombolytic agents delivered by local IA infusion in the first pass of blood following recanalization may be particularly damaging due to the lack of systemic dilution of the thrombolytic agent. Considering this, we closely mimicked such conditions by use of a snare ligature model, which enabled us to initiate IA rt-PA and Δ(K2-K5) plasmin immediately prior to vascular recanalization. Furthermore, as fibrin degradation products may contribute to thrombolytic-associated ischemic damage [38,39], the snare ligature model allowed us to dissociate hemorrhagic damage caused by rt-PA or Δ(K2-K5) plasmin from that caused by degradation products. Thus, the effects on bleeding were intrinsic to the test articles and not a secondary phenomenon. Δ(K2-K5) plasmin even at high dosages infused IA did not result in more severe ICH than saline or vehicle treated animals. This suggests that Δ(K2-K5) plasmin, at the doses used here, did not show an intrinsic tendency to increase ICH, whereas even the lowest dose (accepted thrombolytic therapeutic dose in the rat) of rt-PA did. In addition, there was no evidence of increased bleeding with increasing doses of Δ(K2-K5) plasmin suggesting toxic dosages with respect to bleeding were not reached.

IA administration of rt-PA may be a more effective route for thrombolysis than IV and thus, less drug may be needed [40]. When 10 mg/kg rt-PA was administered to rats IA following 3 hours thromboembolic stroke, there was reduced infarct volume and MRI indicators of damage were improved over the same dose IV without a concomitant increase in ICH [41]. The authors could have titrated the IA dose to match the infarct volume of IV dose but that would have been counterproductive. These data suggest that 10 mg/kg rt-PA IA or IV in the rat is the effective dose. In the clinic, for IA dosing of rt-PA, the total IV dose is typically split; two thirds IV followed by one third IA. Thus the patient receives the complete IV dose even with IA administration. Indeed, there is a current push to administer the complete IV dose of rt-PA and then to supplement with additional rt-PA IA, resulting in an overall dose exceeding the current ceiling of 0.9 mg/kg [42]. This may lead to even greater ICH risk to the patient and may call for severe restriction of patient selection for this treatment paradigm.

IA rt-PA administration, though more effective in lysing a large thrombus in the M1 segment, results in increased risk of ICH compared to IV treatment alone, thereby confirming the extremely narrow safety margin of rt-PA. Furthermore, overdose of rt-PA in the clinic may be more prevalent than previously thought as physicians tend to overestimate the body weight of stroke patients [43]. Δ(K2-K5) plasmin, as an add on to rt-PA treatment or as a stand-alone therapy has the potential to circumvent these complications.

### Infarct volume

Similar to our previous study [11] and consistent with the literature [44,45], the greatest expansion of infarct volume in the SHR occurs within the first 2–3 hours of MCAo, observed in mechanical [11,44] as well as thromboembolic models [45]. The infarct volumes at 2 and 4 hours and in permanently occluded animals are nearly the same suggesting an asymptote effect. However and surprisingly, there was a secondary increase in infarct volume following reflow after 6 hours MCAo. The cause of the spike in infarct volume is unclear but may be related to a significant increase in brain edema. The contralateral to ipsilateral hemisphere ratio was markedly and significantly reduced after 6 hours MCAo/reflow group compared to all other groups. Ischemia predisposes the blood brain barrier to breakdown leading to an increase in vasogenic edema, especially after an extended ischemic event. Permanent MCAo caused a significant increase in edema compared to sham and 1 hour ischemia. Return of blood flow to areas of severe blood brain barrier damage would augment edema formation and thus, may aggravate cerebral infarction.

Our study included 4 control groups: saline, vehicle, and the two rt-PA groups. Statistical analysis comparing these groups suggests that they are statistically significantly the same (i.e. p > 0.95; saline group vs. vehicle group >0.9); reflecting the remarkable consistency of the SHR in this model system, similar to that reported in the literature [23] as well as in our previous study [11]. Considering this consistency, it would not be unreasonable to merge the data from these 4 groups and likewise to do the same with the data from animals treated with Δ(K2-K5) plasmin (consistent with clinical studies [46]). The infarct volume of the resulting control group is 255.3 ± 7.4 (mean ± SEM, n = 23) while that of the resulting Δ(K2-K5) plasmin group is 211.9 ± 9.4 (mean ± SEM, n = 22). Statistical analysis of these two groups is highly significant (p < 0.001, Students *t*-test). This result was unexpected – due to the extended ischemic duration, the infarct volumes of all of the groups were expected to be very similar.

The FDA-approved indirect-acting thrombolytic agent, rt-PA, is an effective means of achieving recanalization therapy. Our temporal data clearly shows that recanalization beyond 2–3 hours is non-therapeutic and thus, rt-PA at any dose past 3 hours post-ictus should not reduce infarct volume in our model system. A priori, there was no expectation of infarct volume reduction for any treatment especially for a thrombolytic agent including Δ(K2-K5) plasmin. Indeed, expansion of cerebral damage, especially after a 6 hour ischemic insult, with high dose rt-PA treatment was more probable and anticipated. Considering the ischemic duration and the use of the SHR, the observed trend for reduction of infarct volume with Δ(K2-K5) plasmin treatment was remarkable.

The reason for the potential improvement in infarct volume is not clear. However, depleting the circulating plasma concentration of α_2_-AP by use of antibodies, plasmin or microplasmin in permanent cerebral ischemia has been shown to reduce infarct volume [47] and/or improve behavior [48]. As Δ(K2-K5) plasmin not bound to fibrin would bind and remove α_2_-AP from the systemic circulation, it also may have neuroprotective properties. On a molar basis, the doses of Δ(K2-K5) plasmin used in our study are within the putative neuroprotection range. Further, because of its smaller size and lack of a requirement for plasminogen, Δ(K2-K5) plasmin may be better than rt-PA at lysing microclots that may become lodged in the periphery of the penumbra thereby improving regional blood flow. These two possibilities may explain the remarkable behavioral recovery of Δ(K2-K5) plasmin treated rats as well as the tendency for reduced infarct volume.

### Behavioral observations

The modified Bederson score is a rough estimate of the functional recovery of the rat and is not sensitive to subtleties. To obtain a better overall judgment of the rats’ recovery, we analyzed the clinical evaluation of the animals examined 2 hours before euthanasia (22 hours post MCAo onset) by a member of the CALS staff (BJW) who had no knowledge of the treatment of the rats. Indeed, the score is simply a numerical value applied to categories already defined by the CALS staff as part of the standard care for animals recovering from surgery. The majority of the animals treated with Δ(K2-K5) plasmin showed significantly greater activity and alertness than the control or rt-PA treated rats. This improved behavior may be an intrinsic property of Δ(K2-K5) plasmin or it may reflect the tendency of Δ(K2-K5) plasmin treatment to reduce infarct volume and to not aggravate ICH. Future studies are needed to better define this observation.

### Model selection

Selection of the snare ligature model allowed us to strictly control the timing of reflow, leading to less variability in the infarct volume data. This is not possible with a thromboembolic model, where the initiation of thrombolytic treatment can be controlled but the timing of recanalization cannot. Our data suggests that an interval as short as 30 minutes can have marked effects on the size of the infarct volume, especially in the early (<4 hours) ischemia time window. Further, in a thromboembolic model, the saline and vehicle control groups would necessarily reflect permanent ischemia and thus no real baseline would be set for test article treatment comparison which may lead to misinterpretation of the data. This is confirmed by our data (Figure 5) showing a significant increase in infarct volume at 6 hours ischemia with reflow compared to the permanently occluded group. Thus, using a thromboembolic model, the control infarct volume used to compare the treatment groups would be 166 ± 10 mm^3^ which would lead to the incorrect conclusion that rt-PA and Δ(K2-K5) plasmin treatment increased infarct volume.

In addition, the snare ligature model allows for the initiation of local IA administration of test article to immediately precede reflow – this is not possible using an intraluminal model where a significant time delay must occur in order to exchange the occluder for the dosing catheter. This is in addition to other complications associated with the intraluminal model such as premature reflow [15], ischemia to the hypothalamus resulting in hyperthermia [16], distension of the MCA (personal observations) and filament related subarachnoid hemorrhage [15].

## Conclusions

The purpose of this study was to assess the ICH safety of the novel direct acting thrombolyic, Δ(K2-K5) plasmin, as a prelude to clinical studies. Even at the highest dose administered, Δ(K2-K5) plasmin caused no worse ICH than the lowest dose of rt-PA but demonstrated at least a 5-fold greater safety margin than rt-PA – potentially making Δ(K2-K5) plasmin a better selection for add-on thrombolytic therapy with rt-PA, or as a stand-alone thrombolytic treatment. Surprisingly, Δ(K2-K5) plasmin showed potential as a neuroprotective agent by virtue of its tendency to reduce infarct volume and improve behavior. Future studies will be required to assess the full potential of Δ(K2-K5) plasmin in stroke.

## Abbreviations

α2AP, α2-antiplasmin; CALS, College of Agriculture and Life Sciences; ECA, External carotid artery; EC-ICA, Extra-cranial internal carotid artery; FDA, Food and Drug Administration; IA, Intra-arterial; ICH, Intracranial hemorrhage; IV, Intravenous; MCA, Middle cerebral artery; MCAo, Middle cerebral artery occlusion; NCSU, North Carolina State University; NOAEL, No observable adverse effect level; rt-PA, Recombinant tissue type plasminogen activator; SHR, Spontaneously hypertensive rat strain; TTC, 2, 3, 5 triphenyl tetrazolium chloride.

## Competing interests

R. Christian Crumrine, G. McLeod Taylor, and Vikram Arora are employed by Grifols Therapeutics, Inc., Research Triangle Park NC. Philip Scuderi, and Stephen Petteway, Jr. are retired from and are consultants for Grifols Therapeutics, Inc. Constantinos P. Tsipis, Joseph C. LaManna and Victor J. Marder are paid consultants for Grifols Therapeutics, Inc. The rt-PA used in the study is commercially available.

## Authors’ contributions

RCC performed the surgeries, was involved in the conception and planning of the experiment and drafted the manuscript. VJM participated in the design of the experiment and edited the manuscript. GMT participated in the execution of the experiment, in the data analysis and in the design of experimental devices. JCL participated in the analysis of data, consulted in the experimental design and provided intellectual input to the drafting of the manuscript. TPS was crucial to the analysis of the infarct volume and Bleeding Score data. VN was responsible for the in vitro experiment. PS, SRP and VA conceived of the experiment and participated in the planning stage. All authors read and approved the manuscript.

## Supplementary Material

Additional file 1 **Table S1.** Pre-ischemia physiological variables, **Table S2.** Pre-reflow physiological variables, **Table S3.** 5 minutes post-reflow physiological variable, **Table S4.** Data graphed in figure 9, **Table S5.** Data graphed in figure 12, **Table S6.** Data graphed in figure 13. (PDF 2006 kb) Click here for file
