# Supplementary material for: Safety evaluation of a recombinant plasmin derivative lacking kringles 2–5 and rt-PA in a rat model of transient ischemic stroke
Source: Exp Transl Stroke Med. 2012 May 16;4:10. doi: 10.1186/2040-7378-4-10 (PMC3464715; doi:10.1186/2040-7378-4-10)
Supplement: Additional file 1 — Table S1. Pre-ischemia physiological variables, Table S2. Pre-reflow physiological variables, Table S3. 5 minutes post-reflow physiological variable, Table S4. Data graphed in figure 9, Table S5. Data graphed in figure 12, Table S6. Data graphed in figure 13. (PDF 2006 kb) [file 2040-7378-4-10-S1.pdf]

Safety evaluation of a recombinant plasmin derivative lacking kringles 2-5 and rt-PA in a rat model of transient ischemic stroke

R. Christian Crumrine, PhD[1]; Victor J. Marder, MD[2]; G. McLeod Taylor, DVM[1]; Joseph C. LaManna, PhD[3]; Constantinos P. Tsipis, BS[3]; Valery Novokhatny, PhD[1]; Philip Scuderi, PhD[1]; Stephen R. Petteway, Jr. , PhD[1]; Vikram Arora, PhD, DABT[1].

[1]Grifols Therapeutics, Inc., Research Triangle Park, North Carolina, USA; [2]David Geffen School of Medicine at UCLA, Los Angeles, CA, USA; [3]Case Western Reserve University, Cleveland, Ohio, USA

Table S1. Physiological Variables – Pre-Ischemia

Pre-Ischemia	Saline (n=6)	Vehicle (n=6)	rt-PA (10 mg/kg; n=5)	rt-PA (30 mg/kg; n=6)	Δ(K2-K5) Plasmin (0.15 mg/kg; n=6)	Δ(K2-K5) Plasmin (0.5 mg/kg; n=6)	Δ(K2-K5) Plasmin (1.5 mg/kg; n=6)	Δ(K2-K5) Plasmin (5 mg/kg; n=5)
pH	7.47 ± 0.01	7.48 ± 0.01	7.47 ± 0.01	7.45 ± 0.01	7.47 ± 0.01	7.47 ± 0.01	7.46 ± 0.01	7.46 ± 0.02
pCO2 (mmHg)	38.0 ± 1.0	37.1 ± 1.2	38.7 ± 0.5	40.2 ± 1.0	37.7 ± 0.9	37.7 ± 1.4	37.9 ± 0.9	37.9 ± 0.0
pO2 (mmHg)	100.3 ± 5.8	109.6 ± 3.6	97.0 ± 5.3	115.8 ± 7.8	105.0 ± 3.4	116.0 ± 5.0	113.5 ± 9.0	97.8 ± 8.7
Glu (mmol/L)	7.0 ± 0.4	7.5 ± 0.5	7.1 ± 0.2	7.7 ± 0.7	7.1 ± 0.1	7.2 ± 0.5	6.5 ± 0.2	6.5 ± 0.2
MABP (mmHg)	134.9 ± 25.2	135.0 ± 14.7	114.3 ± 8.9	170.8 ± 16.7	166.1 ± 12.6	188.6 ± 19.5	163.7 ± 17.0	147.5 ± 14.8
PP (mmHg)	50.5 ± 6.4	52.9 ± 6.7	51.0 ± 1.6	67.3 ± 5.7	61.3 ± 2.0	62.9 ± 8.0	61.4 ± 4.0	49.9 ± 3.7
HR (beats/min)	355 ± 12	355 ± 11	343 ± 6	368 ± 9	343 ± 3	339 ± 9	323 ± 9	341 ± 4
Body Temp (° C)	37.1 ± 0.0	36.9 ± 0.1	37.1 ± 0.0	37.2 ± 0.1	37.1 ± 0.1	37.0 ± 0.0	37.0 ± 4.1	37.0 ± 0.0
Body Wt (g)	363.7 ± 6.1	364.8 ± 5.6	363.5 ± 3.4	362.3 ± 6.9	359.5 ± 4.5	363.5 ± 4.2	356.0 ± 4.1	355.2 ± 7.5
BE (mmol/L)	3.8 ± 0.5	3.8 ± 0.2	4.4 ± 0.8	4.0 ± 0.5	3.7 ± 0.2	4.2 ± 0.7	3.0 ± 0.4	3.4 ± 0.7
HCO3 (mmol/L)	27.4 ± 0.4	27.4 ± 0.2	28.0 ± 0.7	27.8 ± 0.3	27.3 ± 0.3	27.6 ± 0.6	26.8 ± 0.3	27.1 ± 0.6
TCO2 (mmol/L)	28.5 ± 0.4	28.3 ± 0.2	29.0 ± 0.7	29.2 ± 0.3	28.5 ± 0.4	28.7 ± 0.7	27.8 ± 0.3	28.2 ± 0.5
sO2 (%)	97.8 ± 0.5	98.7 ± 0.2	97.6 ± 0.4	98.5 ± 0.2	98.5 ± 0.2	98.8 ± 0.2	98.3 ± 0.4	97.8 ± 0.4
Na (mmol/L)	139.7 ± 0.3	139.8 ± 0.2	139.0 ± 0.5	139.5 ± 0.5	139.7 ± 0.4	139.8 ± 0.6	139.8 ± 0.3	139.6 ± 0.2
K (mmol/L)	4.5 ± 0.1	4.4 ± 0.1	4.5 ± 0.1	4.5 ± 0.1	4.6 ± 0.1	4.5 ± 0.1	4.3 ± 0.1	4.5 ± 0.1
iCa (mmol/L)	1.3 ± 0.0	1.3 ± 0.0	1.3 ± 0.0	1.3 ± 0.0	1.3 ± 0.0	1.3 ± 0.0	1.3 ± 0.0	1.3 ± 0.0
Hct (%PCV)	0.43 ± 0.01	0.46 ± 0.01	0.45 ± 0.01	0.45 ± 0.02	0.47 ± 0.01	0.46 ± 0.01	0.46 ± 0.01	0.46 ± 0.01
Hb (mmol/L)	15.0 ± 0.3	15.8 ± 0.2	15.4 ± 0.2	15.2 ± 0.8	15.8 ± 0.3	15.7 ± 0.2	15.7 ± 0.2	15.7 ± 0.2
Systolic BP (mmHg)	163.7 ± 21.5	166.2 ± 14.4	147.1 ± 9.4	211.7 ± 19.2	198.0 ± 11.4	225.8 ± 19.5	202.8 ± 18.6	172.1 ± 13.5
Diastolic BP (mmHg)	113.2 ± 21.5	113.3 ± 12.6	96.1 ± 8.4	144.4 ± 14.6	136.7 ± 10.3	162.9 ± 18.6	136.5 ± 15.0	122.2 ± 10.8

Rats were prepared for MCAo. Blood from the tail arterial line was analyzed for the indicated variables 5 minutes before MCAo. Values in yellow are significantly different from the corresponding values in Table 5. Values are the mean ± SEM.

Table S2. Physiological Variables: Pre-Reflow

Pre-Reflow	Saline (n=6)	Vehicle (n=6)	rt-PA (10 mg/kg; n=5)	rt-PA (30 mg/kg; n=6)	Δ(K2-K5) Plasmin (0.15 mg/kg; n=6)	Δ(K2-K5) Plasmin (0.5 mg/kg; n=6)	Δ(K2-K5) Plasmin (1.5 mg/kg; n=6)	Δ(K2-K5) Plasmin (5 mg/kg; n=5)
pH	7.44 ± 0.01	7.42 ± 0.01	7.41 ± 0.02	7.43 ± 0.03	7.42 ± 0.01	7.43 ± 0.02	7.40 ± 0.03	7.40 ± 0.02
pCO2 (mmHg)	38.4 ± 0.5	40.3 ± 2.2	38.5 ± 0.6	37.3 ± 0.9	38.7 ± 0.6	38.0 ± 1.3	37.6 ± 1.6	38.3 ± 2.4
pO2 (mmHg)	134.8 ± 6.5	119.3 ± 9.7	144.0 ± 8.2	144.3 ± 6.7	134.8 ± 5.2	136.0 ± 13.4	144.7 ± 10.7	132.2 ± 4.5
Glu (mmol/L)	14.7 ± 2.4	14.2 ± 2.2	23.3 ± 3.7	19.0 ± 3.0	20.0 ± 3.3	19.9 ± 3.6	26.8 ± 4.3	25.7 ± 4.6
MABP (mmHg)	154.2 ± 17.7	132.7 ± 14.7	108.4 ± 3.4	141.2 ± 13.1	137.9 ± 13.1	148.3 ± 12.6	114.4 ± 9.6	159.8 ± 12.6
PP (mmHg)	59.6 ± 6.7	66.0 ± 6.9	67.1 ± 1.9	67.1 ± 5.4	70.1 ± 2.4	74.7 ± 9.2	66.0 ± 5.7	81.4 ± 7.6
HR (beats/min)	362 ± 9	375 ± 12	343 ± 5	367 ± 5	354 ± 6	350 ± 6	339 ± 7	357 ± 14
Body Temp (° C)	37.0 ± 0.0	37.1 ± 0.1	37.1 ± 0.1	37.0 ± 0.0	37.2 ± 0.1	37.1 ± 0.0	37.1 ± 0.0	37.1 ± 0.1
BE (mmol/L)	1.5 ± 0.5	2.3 ± 0.8	0.4 ± 1.6	1.0 ± 2.1	0.3 ± 0.6	0.7 ± 1.2	-1.3 ± 1.3	-0.4 ± 1.2
HCO3 (mmol/L)	25.3 ± 0.6	27.8 ± 1.9	24.9 ± 1.3	25.2 ± 1.7	24.9 ± 0.3	25.1 ± 1.0	23.5 ± 0.7	24.5 ± 0.9
TCO2 (mmol/L)	26.3 ± 0.6	27.3 ± 0.7	25.8 ± 1.3	26.2 ± 1.7	25.7 ± 0.6	26.2 ± 1.1	24.7 ± 0.8	25.8 ± 1.1
sO2 (%)	99.2 ± 0.2	98.5 ± 0.2	99.2 ± 0.2	99.3 ± 0.2	99.0 ± 0.0	99.0 ± 0.3	99.2 ± 0.2	99.0 ± 0.0
Na (mmol/L)	140.3 ± 0.6	140.5 ± 0.9	139.4 ± 1.0	137.8 ± 0.7	139.0 ± 0.7	139.7 ± 0.7	138.3 ± 0.7	138.6 ± 1.3
K (mmol/L)	4.1 ± 0.1	4.2 ± 0.0	4.1 ± 0.1	4.1 ± 0.1	3.9 ± 0.0	3.9 ± 0.1	3.8 ± 0.2	4.3 ± 0.0
iCa (mmol/L)	1.2 ± 0.0	1.2 ± 0.0	1.2 ± 0.0	1.1 ± 0.0	1.2 ± 0.0	1.2 ± 0.0	1.2 ± 0.0	1.2 ± 0.0
Hct (PCV)	0.45 ± 0.01	0.45 ± 0.01	0.46 ± 0.01	0.41 ± 0.02	0.47 ± 0.01	0.47 ± 0.01	0.48 ± 0.01	0.48 ± 0.01
Hb (mmol/L)	15.2 ± 0.3	15.4 ± 0.4	15.7 ± 0.2	14.1 ± 0.8	15.9 ± 0.4	15.9 ± 0.4	16.2 ± 0.3	16.3 ± 0.2
Systolic BP (mmHg)	185.2 ± 17.5	170.1 ± 15.3	149.7 ± 3.6	182.0 ± 12.1	180.1 ± 12.9	193.0 ± 15.9	153.3 ± 12.9	208.6 ± 15.3
Diastolic BP (mmHg)	125.6 ± 15.9	104.1 ± 12.0	82.5 ± 2.7	114.9 ± 12.7	110.1 ± 11.6	118.4 ± 10.5	87.3 ± 7.7	127.2 ± 10.6

Rats were prepared for snare ligature removal. Blood from the tail arterial line was analyzed for the indicated variables 5 minutes before reflow. The significant rise in plasma glucose reflects allowing the rats free access to food between the MCAo and reflow surgeries. Values are the mean ± SEM.

Table S3. Physiological Variables: End of Test Article Infusion

End of Test Article Infusion	Saline (n=6)	Vehicle (n=6)	rt-PA (10 mg/kg; n=5)	rt-PA (30 mg/kg; n=6)	Δ(K2-K5) Plasmin (0.15 mg/kg; n=6)	Δ(K2-K5) Plasmin (0.5 mg/kg; n=6)	Δ(K2-K5) Plasmin (1.5 mg/kg; n=6)	Δ(K2-K5) Plasmin (5 mg/kg; n=5)
Systolic BP (mmHg)	173.4 ± 15.8	160.8 ± 4.7	211.0 ± 9.7	191.8 ± 8.4	179.3 ± 14.6	169.8 ± 16.3	167.2 ± 11.9	204.3 ± 15.0
Diastolic BP (mmHg)	114.4 ± 12.0	99.2 ± 6.3	124.0 ± 10.6	103.6 ± 8.9	111.4 ± 12.3	118.4 ± 10.5	104.0 ± 8.2	125.4 ± 13.0
MABP (mmHg)	138.2 ± 13.8	121.1 ± 5.4	158.5 ± 11.7	137.4 ± 9.4	137.9 ± 13.2	124.0 ± 12.5	130.2 ± 9.4	157.4 ± 14.7
HR (beats/min)	361 ± 10	358 ± 8	356 ± 7	340 ± 6	352 ± 9	346 ± 9	338 ± 7	356 ± 8
PP (mmHg)	59.0 ± 6.1	61.6 ± 5.9	86.9 ± 5.4*	88.3 ± 4.7*	68.0 ± 4.5	72.3 ± 7.8	63.2 ± 5.5	78.8 ± 6.5

* $p<0.05$ compared to Pre-Reflow values (t-test). PP is pulse pressure

Table S4, S5 and S6. Data presented in Figures 9, 12 and 13, respectively

S4. Bleeding Score (Figure 9)

Group	Median	5%	95%
Saline	1.5	1	2.5
Vehicle	1.5	0	2.4
10 mg/kg rt-PA	3	1.5	4.5
30 mg/kg rt-PA	4.75	2	5
0.15 mg/kg Δ(K2-K5) plasmin	2.25	1	3
0.5 mg/kg Δ(K2-K5) plasmin	2.25	2	4
1.5 mg/kg Δ(K2-K5) plasmin	1.75	1	2.5
5 mg/kg Δ(K2-K5) plasmin	2	1	3

S5. Modified Bederson Score (Figure 12)

Saline	2	2	3
Vehicle	2	1.33	2.33
10 mg/kg rt-PA	2.33	2	3.33
30 mg/kg rt-PA	3.33	2.33	4
0.15 mg/kg Δ(K2-K5) plasmin	2.16	1	2.33
0.5 mg/kg Δ(K2-K5) plasmin	1.67	1.33	2
1.5 mg/kg Δ(K2-K5) plasmin	2	1.33	2.33
5 mg/kg Δ(K2-K5) plasmin	2.33	2	2.33

S6. General Behavioral Score (Figure 13)

Saline	1	1	2
Vehicle	1.25	1	2
10 mg/kg rt-PA	1	1	3
30 mg/kg rt-PA	3	2.5	5
0.15 mg/kg Δ(K2-K5) plasmin	0.75	0.5	1
0.5 mg/kg Δ(K2-K5) plasmin	1	0.75	1
1.5 mg/kg Δ(K2-K5) plasmin	1	0.75	2
5 mg/kg Δ(K2-K5) plasmin	0.75	0.5	1.25
